# Effects of supplementation with a blend of trisodium citrate, creatine monohydrate, leucine, and blueberry extract on training-induced changes in leg extension strength, endurance, and muscle size

**DOI:** 10.1080/15502783.2025.2550141

**Published:** 2025-08-25

**Authors:** Trevor D. Roberts, Jocelyn E. Arnett, Dolores G. Ortega, Justin S. Pioske, Joseph F. Daugherty, Michael S. Tempesta, Alekha K. Dash, Richard J. Schmidt, Terry J. Housh

**Affiliations:** a University of Nebraska-Lincoln, Department of Nutrition and Health Sciences, Exercise Physiology Laboratory, Lincoln, NE, USA; b Phenolics LLC, Omaha, NE, USA; c School of Pharmacy and Health Professions, Department of Pharmacy Sciences, Omaha, NE, USA

**Keywords:** Hypertrophy, sports nutrition, Supplement, Performance

## Abstract

**Background:**

This study examined the effects of 8 weeks of supplementation with a blend of trisodium citrate, creatine monohydrate, leucine, and blueberry extract (TCLB) during resistance training on leg extension (LegExt) strength, LegExt endurance, and quadriceps muscle size.

**Methods:**

A randomized, double-blind design was used to compare the effects of TCLB to creatine monohydrate (CM) and placebo (PLA). Twenty-eight recreationally active men (mean ± SD: age = 20.6 ± 1.4 yr; body mass = 86.9 ± 11.6 kg) ingested either TCLB (*n* = 10), CM (*n* = 10), or PLA (*n* = 8) during 8 weeks of LegExt resistance training. The following assessments were conducted before and after the training: 1) LegExt 1-repetition maximum (1RM), 2) LegExt repetitions-to-failure at approximately 80% of pre-training 1RM, 3) cross-sectional area (CSA) values of the individual quadriceps muscles (rectus femoris [RF], vastus lateralis [VL], vastus intermedius [VI], and vastus medialis [VM]), and 4) the sum of the CSA values (CSA_sum_ = RF + VL + VI + VM). Separate one-way ANCOVAs that were covaried for pre-training values were used to analyze differences in the adjusted post-training means, and dependent *t*-tests with Bonferroni corrections were used to assess post-hoc pairwise comparisons. Chi-squared tests were used to evaluate differences between groups in the proportion of subjects who exceeded the minimal important difference (MID = pooled SD × 0.5).

**Results:**

The ANCOVAs indicated no significant (*p* = 0.110–0.542) group differences in the adjusted post-training means for LegExt 1RM, LegExt repetitions-to-failure, RF CSA, VL CSA, or VM CSA. The ANCOVAs for VI CSA (*p* = 0.002) and CSA_sum_ (*p* = 0.006) were significant. Pairwise comparisons indicated that the TCLB group (VI CSA = 33.37 cm^2^; CSA_sum_ = 99.09 cm^2^) exhibited greater (*p* = 0.001–0.005) adjusted post-training means for VI CSA and CSA_sum_ than the PLA group (VI CSA = 31.64 cm^2^; CSA_sum_ = 95.37 cm^2^). The Chi-square tests showed no significant (*p* = 0.136–0.867) differences in the proportion of subjects exceeding the MID for LegExt 1RM, LegExt repetitions-to-failure, RF CSA, and VM CSA across all group comparisons. The TCLB group (VI CSA = 40%; CSA_sum_ = 60%) demonstrated a greater (*p* = 0.007–0.043) proportion of subjects exceeding the MID for VI CSA and CSA_sum_ than the PLA group (VI CSA = 0%; CSA_sum_ = 0%). The CM group (40%) exhibited a greater (*p* = 0.043) proportion of subjects exceeding the MID for VL CSA than the PLA group (0%).

**Conclusions:**

Eight weeks of supplementation with a blend of TCLB during resistance training increased total quadriceps muscle size more than the PLA, primarily from enhanced increases in the size of the VI muscle, without affecting training-induced changes in LegExt strength and endurance.

